# Retrospective assessment of ICD-10/DSM-5 criteria of childhood ADHD from descriptions of academic and social behaviors in German primary school reports

**DOI:** 10.1007/s00787-024-02509-4

**Published:** 2024-07-24

**Authors:** Johanna Waltereit, Martin Schulte-Rüther, Veit Roessner, Robert Waltereit

**Affiliations:** 1 Department of Child and Adolescent Psychiatry, University Medical Center Göttingen, Von-Siebold-Str. 5, 37075 Göttingen, Germany; 2 Department of Child and Adolescent Psychiatry, Medical Faculty Carl Gustav Carus, TU Dresden, Dresden, Germany; 3 Department of Child and Adolescent Psychiatry, LWL-Klinikum Marsberg, Marsberg, Germany; 4 Department of Child and Adolescent Psychiatry, Center for Psychosocial Medicine - University Hospital Heidelberg, Ruprecht-Karls-University Heidelberg, Heidelberg, Germany

**Keywords:** Childhood ADHD, Retrospective analysis, School reports, Diagnostic process, Adolescents and adults

## Abstract

**Background:**

The diagnosis of attention-deficit/hyperactivity disorder (ADHD) in adolescence and adulthood is particularly challenging because retrospective confirmation of previous childhood ADHD is mandatory. Therefore, collecting valid diagnostic information about behavior at school is important. Primary school reports often contain descriptions of academic performance and social behaviors associated with ADHD criteria. Yet, there is no systematic approach available how to assess such reports quantitatively, and therefore, there is also no study on how valid such an approach could predict an ADHD diagnosis.

**Methods:**

We examined primary school reports from Germany (ADHD: *n* = 1197, typically developing controls: *n* = 656) for semantic references to ICD-10/DSM-5 main and sub-criteria of ADHD. Descriptions were assessed on a quantitative scale (blinded clinical expert rating) for disorder-associated behaviors (symptoms scale) as well as for desired, adaptive behaviors (competencies scale) according to these criteria. The scores of these developed scales have been summarized to summary scores. Scores were analyzed using linear mixed models, and sensitivity and specificity were estimated using receiver operating characteristics (ROC).

**Results:**

Ratings showed highly significant differences between school reports of children with and without ADHD. For the summary scores, both symptoms and competencies scales showed high diagnostic accuracy (ROC area under the curve at least 0.96) with best discrimination when combining both into an integrated index (sensitivity and specificity > 0.97).

**Conclusions:**

Our findings suggest that systematic quantitative analysis of primary school reports should be further explored to construct a valid instrument for retrospective assessment of childhood ADHD criteria to aid the diagnostic process in adolescents and adults.

**Supplementary Information:**

The online version contains supplementary material available at 10.1007/s00787-024-02509-4.

## Introduction

The current study examined how descriptions of academic performance and social behaviors in primary school reports can be used to retrospectively assess criteria of attention-deficit/hyperactivity disorder (ADHD) quantitatively and how to predict the diagnosis of childhood ADHD. ADHD is a childhood-onset neurodevelopmental disorder, characterized by developmentally inappropriate and impairing inattention, motor hyperactivity and impulsivity, with impairments often persisting into adulthood [1]. Symptoms include a wide range of difficulties in cognitive and social functioning as well as adaptive behavior resulting e.g. in reduced school achievements, impaired personal relationships with peers and teachers and less discipline in classrooms. The diagnostic categories of “ADHD” as defined in the DSM-5 [2] are broadly equivalent to the categories of “hyperkinetic disorder”, as defined in the ICD-10 [3]. ADHD is common, with a pooled worldwide prevalence of around 5.3% in school-aged children [4–6] Symptom severity declines during adolescence [7, 8], therefore the prevalence in early adulthood is about 2.5% [9–11]. Higher prevalence estimates are described in adulthood when the diagnostic challenge of retrospective diagnosis of childhood ADHD is not required [12]. In ICD-10, symptoms need to be present in all three domains of inattention, hyperactivity and impulsivity, while DSM-5 merges hyperactivity and impulsivity into one domain.

The diagnosis of ADHD in children, adolescents and adults is based on a full clinical and psychosocial assessment, including present behavior and symptoms in everyday life, current mental state, as well as developmental and psychiatric history. A diagnosis of ADHD cannot be made solely on the basis of self-rating scales or of observational data and so far. No neurobiological marker or psychometric test has been developed to substitute best-estimate clinical evaluation [12–14].

### Problem description

While ADHD can be reliably diagnosed in childhood [12, 14], this is considerably more difficult in adolescence and adulthood. The diagnostic criteria require the appearance of core symptoms before the age of seven (ICD-10) or before the age of twelve (DSM-5), thus, retrospective assessment of childhood ADHD symptoms is mandatory [1], and a key issue in diagnostic assessment of ADHD in adolescents and adults [12, 14, 15].

Retrospective information about ADHD core symptoms in childhood can either be obtained from (1) self-reports, (2) parent /caregiver reports or, (3) reports of institutional observers (e.g. teachers). Retrospective reports of all three approaches rely – at least in parts – on potentially biased subjective memories. Methodologically, information about ADHD symptoms of one individual can be obtained from each of these three groups of reporters/informants by different tools such as clinical diagnostic interviews, rating scales and subjective/objective (observer) reports.

Addressing the first group of informants, a clinical diagnostic interview with an adolescent or adult patient has limited value in this context [16]. Self-rating instruments to retrospectively assess childhood ADHD symptoms in adults have been developed, e.g. the Wender Utah Rating Scale (WURS) and its short forms [17–21]. However, diagnoses based solely on retrospective self-report have been criticized to have poor validity in the majority of cases [22–24]. Consequently, the diagnostic validity of WURS and its short forms has been questioned [24–28].

To assess child development and psychopathology retrospectively from the second group of informants, the clinical diagnostic interview with parents or caregivers is on first glance an established technique. While this technique is a feasible approach in general scenarios to make an actual diagnosis [29], it has not been proven for the specific task of retrospective diagnosing of childhood ADHD in adolescents and adults [15]. In particular, parents of adolescents and young adults with ADHD were found to have limited ability to accurately recall childhood symptoms [23]. Additionally, while several rating scales available for parents’ and caregivers’ information [29–35] have proven their validity to assess the current symptomatology of ADHD in adolescents and adults, their ability to secure childhood ADHD symptoms retrospectively of similar validity has not been demonstrated [15].

Finally, addressing the third group of informants, to obtain retrospective information about childhood ADHD symptoms of an adolescent or even adult patient for example from a teacher is even more difficult and of even more questionable validity. After years the teacher may not be available, and the memory of the teacher – if he has been located at all and has agreed to participate in the assessment - may be questioned for its validity similar to other groups of informants. In contrast, school reports contain important retrospective and temporally defined information from teachers for each year in school and could be used for the purpose of retrospective assessment of childhood ADHD [13, 36]. However, there is no systematic approach available how to assess such reports quantitatively, and therefore, there is also no study on how valid such an approach could predict an ADHD diagnosis. Taken together, there is an urgent need for a more objective, quantitative and valid retrospective assessment of childhood ADHD symptoms. Here, we propose a novel approach which eliminates the above mentioned potential bias resulting from retrospective long-term memory by leveraging primary school reports as fixed, temporally defined observer accounts.

### Descriptions of symptoms and of competencies in German school reports

The diagnostic concept of ADHD includes observable cognitive and social behavior with a focus on school contexts, including primary schools. The ICD-10 and DSM-5 symptoms of inattention, hyperactivity and impulsivity are similar across both nosological systems and mainly concern performance in school but also behavior at home or during leisure time [2, 37, 3]. Consequently, clinical guidelines recommend the assessment of teachers’ information about the child’s academic performance and behavior at school or in written school reports [13, 38]. School reports give structured insights into academic performance, social functioning and adaptive behavior within a temporally defined period of everyday school life that is/was not too long ago from the time of assessment. They are written by teachers who are experts for this evaluation [36, 38–40]. Thus, they offer a currently untapped potential to develop a clinical instrument for valid retrospective assessment of ADHD symptoms.

In this study we used German primary school reports (which have a high level of standardization and detailed specifications due to the guidelines of the respective ministry of culture and education) to demonstrate the feasibility of our approach. In order to compare children with ADHD to typically developing children in an evidence-based way, quantitative assessment (i.e. operationalization) of descriptions in school reports was necessary. However, the methodology should be applicable to any collection of sufficiently detailed standardized reports. To our knowledge, there is no systematic approach available how to quantitatively assess ICD-10 or DSM-5 criteria of ADHD in school reports and no study on how valid such an approach could predict an ADHD diagnosis.

Most psychiatric diagnoses have symptoms that are defined as deviations or dysregulations of behavior or mental processes. Symptoms are often manifestations of patterns of thought, emotion, or behavior that deviate from what is considered typical or healthy resulting in impairments in the level of functioning. Thus, ICD-10/DSM-5 descriptions of ADHD are only descriptions of deviations or dysregulations – “symptoms” - from the perspective of what is considered typical or healthy. The teacher who writes the description of academic performance and social behaviors has to follow also another additional approach. Assessing attentiveness, activity and impulse control in school children, the teacher does not only describe deviations or dysregulations (“symptoms” in view of the clinician) – like inattention, hyperactivity and impulsivity – but also “competencies” – like explicitly good attention, well-adapted activity and control of inappropriate impulses.

Taken together, the aim of this study was to examine with quantitative methods how descriptions of academic performance and social behaviors in primary school reports – both symptoms and competencies - can be used to quantitatively assess ICD-10 or DSM-5 criteria of ADHD retrospectively and how validly they predict the diagnosis of childhood ADHD.

### Hypotheses and research questions

Specifically, we aimed to address two research questions: First, we investigated whether German primary school reports can be used for a valid quantitative assessment of retrospective childhood ADHD symptoms and competencies, according to the main criteria and subcriteria of ADHD as defined in ICD-10 and DSM-5. Second, we determined the diagnostic accuracy of such quantitative assessment, and the feasibility to establish cut-off scores for both sensitive and specific retrospective screening for the presence of childhood ADHD.

We predicted overall group differences between school reports of patients with ADHD compared to typically developing control persons regardless of the respective school year. In addition, the effects of school year and each main and subcriterion of ADHD on group differences were investigated exploratively. We had the hypotheses that school reports of patients with ADHD contain significantly more descriptions of ADHD in contrast to the school reports of typically developing control persons.

## Methods

### Participants

Data were collected from school reports of patients with ADHD and of typically developing control participants, with an intelligence quotient between 70 and 130. All participants were former students at primary schools in the German federal state of Saxony. The sample is described in detail in Table 1. In Supplementary Fig. 1, additional information about the school reports is provided.

**Table 1:** Description of the sample. Three subtypes of ADHD were encoded according to ICD-10: hyperkinetic disorder without conduct disorder (F90.0), hyperkinetic disorder with conduct disorder (F90.1) and the predominantly inattentive subtype (F98.8)

		Typical development	ADHD	F90.0	F90.1	F98.8	unit
**Students**		84	270	143	72	55	n
**Primary school certificates per student**		7.37	7.53	7.58	7.45	7.52	mean
**Sex**		0.452	0.700	0.692	0.778	0.618	ratio male/female
**IQ scales** **HAWIK IV**	**Full Scale IQ**	110	101	101	96	105	mean
	**Verbal Comprehension Index (VCI)**	137	103	102	99	110	mean
	**Fluid Reasoning Index (FRI)**	134	104	103	99	111	mean
	**Working Memory Index (WMI)**	117	96	95	97	98	mean
	**Processing Speed Index (PSI)**	103	95	97	94	93	mean
**School in Saxony**		0.91	0.99	0.99	1	1	ratio
**Type of primary school**	**Regular primary school**						percent
	**1st grade**	90,1%	83,6%	87,9%	77,8%	80,3%	
	**2nd grade**	90,1%	79,5%	85,9%	70,4%	76,8%	
	**3rd grade**	90,1%	77,1%	81,1%	65,6%	75%	
	**4th grade**	89,0%	64,5%	79,8%	62,1%	76,9%	
	**Private primary school (bilingual schools, reform pedagogical schools, schools with focus on sports or music or christian schools)**						percent
	**1st grade**	9,9%	7,4%	5,7%	5,5%	14,3%	
	**2nd grade**	9,9%	7,1%	5,6%	4,2%	14,3%	
	**3rd grade**	9,9%	8,3%	8,33%	4,7%	14,3%	
	**4th grade**	11,0%	9,2%	8,5%	8,6%	13,4%	
	**Primary school for special needs**						
	**1st grade**	0,0%	8,9%	6,4%	16,6%	5,3%	
	**2nd grade**	0,0%	13,4%	8,4%	25,4%	8,9%	
	**3rd grade**	0,0%	14,6%	10,6%	29,7%	10,7%	
	**4th grade**	0,0%	13,2%	11,6%	29,3%	9,6%	
**Secondary school**	**“Gymnasium” (12 years)**	81,8%	21,9%	23,3%	10,6%	31,7%	percent
	**High school (10 years)**	17,0%	39,1%	37,3%	42,5%	29,0%	
	**Middle school (9 years)**	0,0%	25,0%	28,0%	23,4%	17,1%	
	**Private secondary school**	1,1%	1,6%	0,0%	0,9%	4,8%	
	**Secondary school for special needs**	0,0%	12,5%	11,2%	21,3%	7,3%	
**Repeating of a class**		0	0.119	0.112	0.167	0.0727	ratio

Patients included in the study were former or current patients of the inpatient and outpatient departments of Child and Adolescent Psychiatry, University Hospital Carl Gustav Carus, Dresden. They had received or confirmed a diagnosis of ADHD (ICD-10 F90.0), hyperkinetic conduct disorder (ICD-10 F90.1) or attention deficit disorder without hyperactivity (ICD-10 F98.8) at any time between the ages of 12 and 17. All diagnoses were made during regular treatment by board-certified specialists in child and adolescent psychiatry. A clinical best-estimate diagnosis of ADHD was either confirmed or excluded following established guidelines of “gold standard” diagnostic evaluation of ADHD [12–14, 38]. Similarly, co-occurring ICD-F diagnoses were secured. Exclusion criteria were the presence of other psychiatric or neurological disorders than ADHD (ICD-10 chapters F and G). School reports were available at this diagnostic stage but were only used for a coarse impression building from a given patient. The systematic process of semantic school report analysis was performed at least weeks, in many cases years, later by the research department team using the newly developed procedure following ICD-10/DSM-5 criteria. This procedure was in no way involved in or part of the previous diagnostic decision.

Typically developing control persons were selected from a community-based pool of volunteers registered in Dresden for research study participation. They had never received a psychiatric or neurological diagnosis (ICD-10 chapters F and G) by their pediatrician, general practitioner or any other healthcare professional. The absence of a psychiatric or neurological history was additionally checked by a clinical diagnostic interview obtained from parents before inclusion by the study team.

All primary study data for this investigation (sociodemographic data, primary school reports, IQ assessment, medication) were taken from the files of patients at Child and Adolescent Psychiatry, University Hospital Carl Gustav Carus, Dresden, or the files of control persons, who had volunteered in study participation. All primary data included in this study were anonymized at the source to fulfill requirements by the Ethics Committee. The study had been approved by the Ethics Committee of the University Hospital Carl Gustav Carus Dresden (reference number EK 295,072,016).

### Measures

In most countries, primary school reports are standardized documents that contain grades and descriptions on academic performance and social behaviors. In Germany, the main teacher responsible for the respective class writes the obligatory description part of the primary school report, that describes academic and social behavior for each student. His proposal is then discussed and finally approved by a school conference (consisting of student representatives, parent representatives and teachers). Such reports are available at the end of each school year, in most cases also at each half-year term.

The content requirements of the obligatory description part of the German primary school reports as defined by the respective ministry by the ministries of culture and education are as follows: motivation and cooperation, focus on performance and results, ability to cooperate, autonomy, diligence and perseverance and reliability. The content requirements for the evaluation of social behavior are: ability to reflect, ability to engage in conflicts, agreement and compliance with rules and fairness, helpfulness and respect for others, taking responsibility and active participation in the school community. These descriptions of students’ behavior can be supplemented by a description of the students’ skills, abilities and interests. With regard to ADHD, especially the evaluation of academic behavior suggests an overlap with the clinical assessment of ADHD core symptoms.

Students of German primary schools in Saxony receive such an obligatory description of academic performance and social behaviors at the end of each school year, as part of their school report. In the 1st and 2nd year an additional mandatory description is issued with their midterm school report. From the 3rd year onwards, descriptions of academic and social behavior in midterm reports are optional, the yearly descriptions are less detailed, and marks are included for academic performance as well as marks for social behaviors.

These description parts of the primary school reports report on the one hand optimally adapted attentive, controlled and calm behaviors (which are to be evaluated as competencies) and on the other hand highly troublesome inattentive, impulsive and hyperactive behaviors (which are to be evaluated as (sub-) clinical symptoms). Accordingly, in the semantic analysis of primary school reports, descriptions of students’ academic performance and social behaviors, showing a relationship with ICD-10 or DSM-5 criteria of ADHD and classified as (sub-) clinical symptoms or as complementary competencies, were analyzed by experienced clinician raters (psychotherapists working in child and adolescent psychiatry), who were blind to the ADHD diagnosis of the respective child. Interrater reliability (weighted Cohen’s Kappa) for each (sub-) criterion was calculated across all subjects and time points, grouped by item and valence using squared weights. Each Kappa calculation utilized all available paired ratings per item, per valence category, ensuring a robust estimation of agreement between raters (193 rating points available from two raters for each item). Interrater reliability was excellent for most items (between 0.759 and 1, average: 0.901 for symptoms scale, 0.917 for competencies scale). One overall main score was evaluated for the core criterion “hyperactivity” and one for “impulsivity” each comprising all respective subcriteria, because initial screening revealed that descriptions were not detailed enough to give scores for each respective subcriterion. The approach for the core criterion of “inattention” was quite different: Due to a sufficient amount of relevant information in school reports, it was possible to evaluate each of the nine subcriteria separately.

As mentioned before, students’ academic and social behavior can be classified into (sub-) clinical symptoms and into competencies. Since ICD-10/DSM-5 only describes impaired behaviors as main criteria and subcriteria in more detail regarding ADHD symptoms, we have decided to create a scale for “competencies” and a scale for “symptoms” for each (sub-) criterion. The symptoms scales in our study semantically correspond to the exact ICD-10/DSM-5 criteria and are a conceptualization of both “meeting the expectations of teachers with limitations” or “not meeting expectations” according to the guidelines of the Saxon ministry of culture and education, as well as the occurrence of substantial inattentiveness, hyperactivity, and impulsivity. The competencies scales broadly correspond to the conceptualization of the “spectrum of behavior of typically developing children” with respect to academic and social behaviors - attentiveness and age-appropriate adapted behaviors in movement and interaction - in the school context [40]. Criteria were rated on the symptoms scales with a score of 0 when the feature was not mentioned, a score of 1 when subclinical aspects were evident (roughly corresponding to “meeting expectations with limitations”), and a score of 2 when clinical aspects were evident (roughly corresponding to “not meeting expectations of teachers”, “no change in behavior expected in the foreseeable future”). Respective ICD-10/DSM-5 criteria were rated on the competencies scales with a score of 1 for descriptions indicating that the adapted behavior “corresponded to the expectations of teachers” or was even “above expectations”, and were rated with a score of 0 when the respective feature was not mentioned.

Please refer to Supplementary Table 1 for a checklist that was used to assess primary school reports for this study. Supplementary Table 2 contains several examples how ratings of symptoms and competencies were performed.

### Calculation of scores

For each school report eleven scales were rated according to exact ICD-10/DSM-5 criteria (i.e. nine subcriteria scales of the main criterion of inattention and a single overall scale for hyperactivity and impulsivity, respectively). These eleven scales were determined for both symptoms and competencies (i.e. 22 scales in total). Scores for a single school report were 0, 1 or 2 for symptoms (0 = not mentioned, 1 = subclinical, and 2 = clinical symptom expression) and 0 or 1 for competencies (0 = not mentioned, 1 = competencies). Thus, if there were no descriptions defining of a (sub-) criterion scale in a single school report, the respective score was 0, for both symptoms and competencies. If there were two or more descriptions defining a (sub-) criterion scale in a single school report, a single positive score was determined for symptoms or competencies, respectively.

**Fig. 1:**
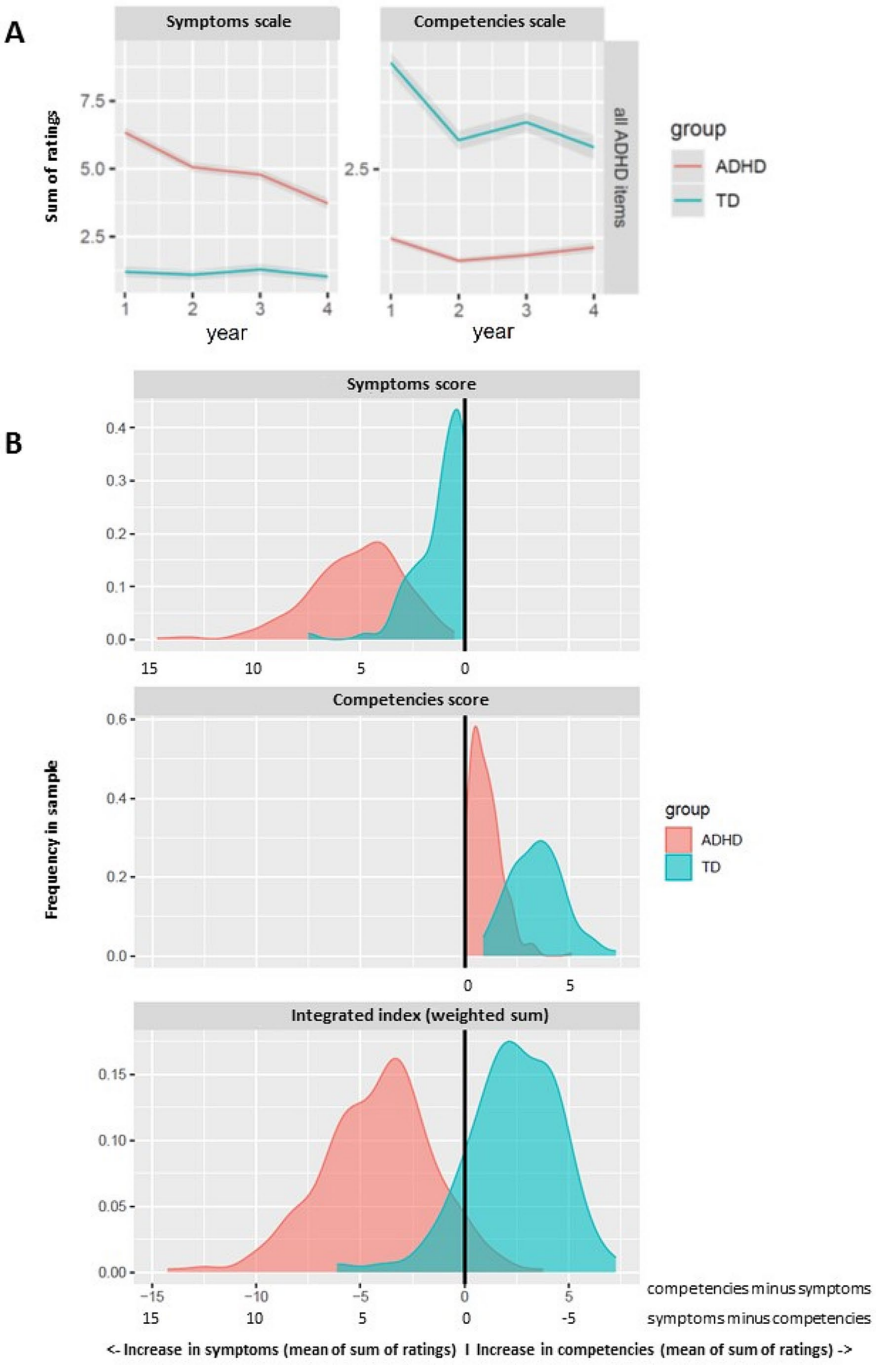
Summary scores of ICD-10/DSM-5 criteria of ADHD described in primary school reports. **(A)** Summed ratings of all ADHD ICD-10/DSM-5 symptoms and competencies. Abscissa: school year (n). Ordinate: sum of all sub-criteria ratings of ICD-10/DSM-5 symptoms of inattention (refer to Fig. 3) and of ratings of ICD-10/DSM-5 main symptoms of hyperactivity and impulsivity (refer to Fig. 2**B**), and of competencies, respectively. Colored lines indicate group means and shaded ribbons indicate the standard error of means. **(B)** Sums of all sub-criteria ratings of ICD-10/DSM-5 symptoms of inattention and of ratings of ICD-10/DSM-5 main symptoms of hyperactivity and impulsivity, and of competencies, respectively. Red color: children with ADHD, blue color: typically developing children. Upper panel: symptoms. Middle panel: competencies. Lower panel: integrated index. Abscissa: means of sums (n). Ordinate: frequency (n)

**Fig. 2:**
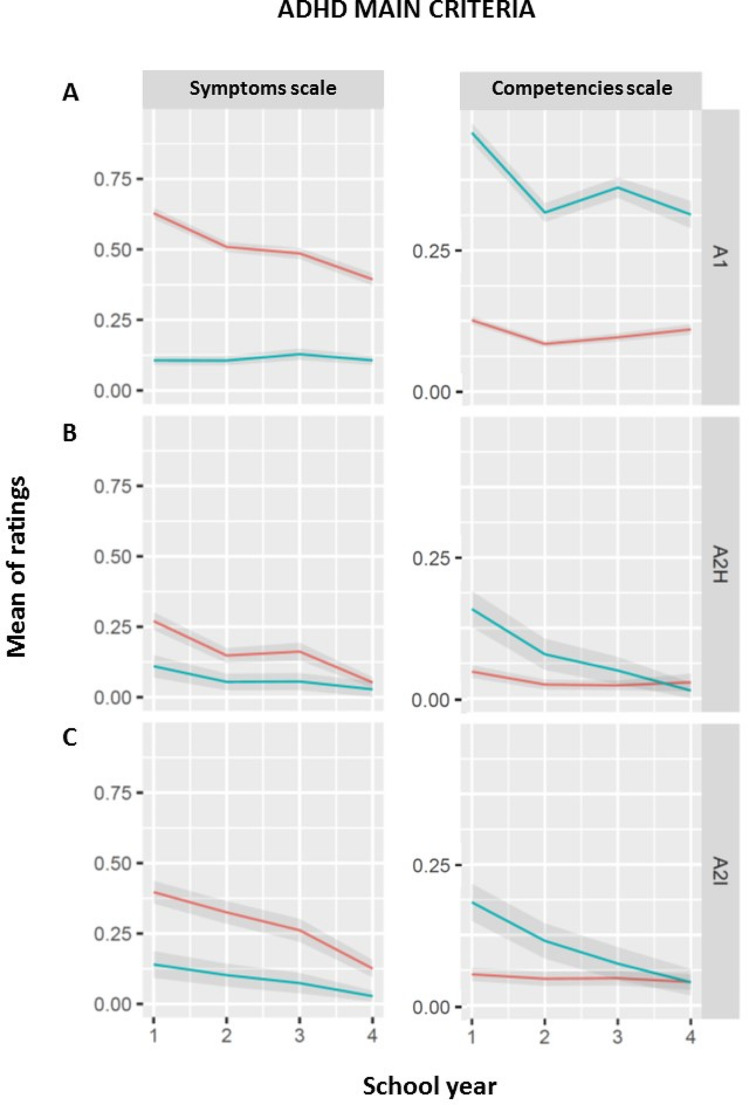
ADHD main criteria in primary school reports. The mean values of all ICD-10/DSM-5 main symptoms of inattention, hyperactivity and impulsivity are shown on the symptoms scale and of the complementary competencies on the competencies scale. **(A)** A1 = inattention, **(B)** A2H = hyperactivity and **(C)** A2I = impulsivity. Abscissa: school year (n). Ordinate: mean of operationalization of ICD-10/DSM-5 main symptoms. For symptoms scale, descriptions of 0 = no symptom in a school report according to semantic analysis, 1 = sub-clinical symptom expression, 2 = clinical symptom expression. For competencies scale, descriptions of 1 = expressions of typically developed behavior complementary to the ADHD symptom, 0 = feature not mentioned. Red line: children with ADHD, blue line: typically developing children. Colored lines indicate group means and shaded ribbons indicate the standard error of means

Overall summary scores (for both symptoms and competencies scales, respectively) were calculated as the sum of ratings of the nine subcriteria of inattention and of the two main criteria hyperactivity and impulsivity (Fig. 1). Additionally, a specific summary score for inattention was calculated as the mean value of score of the nine subcriteria of inattention (Fig. 2A).

### Data analyses

Ratings for each of the symptoms and competencies scales (nine for the subcriteria of inattention and two for the main criteria of hyperactivity and impulsivity, respectively) were summarized within school years (i.e. averaged if two reports were available for one school year) and further analyzed. We calculated summary scores per school year by aggregating hyperactivity, impulsivity, and all inattention subcriteria scales. We then computed final summary scales for each individual across all school years, separately for competencies and symptoms i.e. summary competencies scales, and summary symptoms scales. Additionally, we calculated an integrated index for each participant integrating the entire spectrum of competencies and symptoms. This was achieved by combining the competencies and symptoms scores into a single weighted sum, assigning a weight of -1 to symptoms and a weight of + 1 to competencies, reflecting the dual nature of behavioral expressions observed in classroom settings. Both summary scores and the integrated index were used to calculate the area under the receiver operating characteristic (ROC) curve (AUC), serving to differentiate ADHD from non-ADHD. Additionally, DeLong test for correlated ROCs were performed to compare the respective ROC curves for differences. Lastly, we calculated optimal cut-off scores across the whole sample that maximized the Youden index (sensitivity + specificity − 1) for a balanced optimization of specificity and sensitivity for all three types of scales. To provide an estimate of potential clinical utility for different cut-off scores, we calculated optimal scores that maximized sensitivity for a given specificity of at least (a) 0.90, (b) 0.95, (c) 0.99. Sensitivity, specificity, and Youden index were calculated for each of these cut-off scores, separately for symptoms and competencies scales and integrated index. The R-package cutpoint was used for these analyses.

For an in-depth analysis of the influence of school year and group on summed scores, we performed linear mixed model analyses using the packages lme4 and lmerTest implemented in R 4.3. Group and school year were used as predictors and the ADHD scale as the dependent variable. In an iterative procedure [41], we initially included all random slopes and respective interactions in models with correlated and uncorrelated random effects. Insignificant variance components were removed, and the best model was selected using iterative removal of variance components. The best fitting model did only include “participant” as a random effect, modelling of random slopes and correlated effects did not add significant explanation of variance and were thus discarded. This initial model selection procedure was performed on the integrated index and the final model was applied to all other analyses. Our main analysis was on integrated index, reflecting the full spectrum of behavior. We tested the following linear hypotheses (using Chi-Square Wald tests to determine significance): (1) overall group difference across school years, (2) linear effect of school year on group difference scale (3) group differences separately for each school year, and (4) pairwise comparisons of the first school year against all other years. For (1) and (2) no correction for multiple testing was applied because these hypotheses are statistically independent. For (3) and (4) *p*-value correction was applied, taking into account *n* = 4 school years and *n* = 3 pairwise comparisons, respectively (Bonferroni-Holmes correction). On an exploratory note, these analyses were also performed separately for the summary symptoms score, summary competencies score, and on the subscales of inattention, hyperactivity and impulsivity (no additional *p*-value correction applied for these analyses).

## Results

### Summary scores of ICD-10/DSM-5 criteria of ADHD

Our approach revealed robust group differences between children with ADHD and typically developing children. The integrated index, combining inattention, hyperactivity, and impulsivity from the symptoms scale and the competencies scale, revealed high discriminative potential with only limited overlap between patients with ADHD and typically developing children (see Fig. 1 for group averages of overall ADHD scales and distribution density plots). With respect to the integrated index, highly significant effects of group were evident (i.e. higher symptoms scores and integrated index, and lower competencies scores for those with a diagnosis of ADHD, χ^2^_integrated_(1) = 416.09, *p* < 0.001, χ^2^_comp_(1) = 253.48, *p* < 0.001; χ^2^_symp_(1) = 472.45, *p* < 0.001). Group effects decreased linearly with school year (linear contrast across school years: χ^2^_integrated_(1) = 47.40, *p* < 0.001, χ^2^_comp_(1) = 39.431, *p* < 0.001; χ^2^_symp_(1) = 29.28, *p* < 0.001), but were still significant separately for each school year (all χ^2^_integrated_(1) > 106.2, p_corr_. < 0.001, χ^2^_comp_(1) = 117.96, p_corr_ <0.001; χ^2^_symp_(1) > 59.13, p_corr_ < 0.001). The effects of group were largest in the first school year as compared to all other school years (pairwise linear comparison contrasts, all χ^2^_integrated_(1) > 21.79all p_corr_. < 0.001, all χ^2^_comp_(1) = 16.69, p_corr_ < 0.001; χ^2^_symp_(1) > 9.23, p_corr_ < 0.01).

For the subscales of inattention, impulsivity, and hyperactivity (Fig. 2) the overall effect of group across school years was highly significant for the integrated index (χ^2^_inattention_(1) = 444.70, *p* < 0.001, χ^2^_impulsivity_(1) = 23.85, *p* < 0.001; χ^2^_hyperactivity_(1) = 14.159, *p* < 0.001). This effect decreased linearly with school year (linear contrast across school years 1 to 4: χ^2^_inattention_(1) = 42.25, *p* < 0.001, χ^2^_impulsivity_(1) = 7.071, *p* < 0.01; χ^2^_hyperactivity_(1) = 9.840, *p* < 0.001). For inattention, the effect was also significant separately for each school year (all χ^2^_inattention_(1) > 118.36, p_corr_. < 0.001; χ^2^_n_(1) > 59.13, p_corr_ < 0.001) and was largest in the first school year as compared to all other school years (pairwise linear comparison contrasts, all χ^2^_inattention_(1) > 20.063, all p_corr_. < 0.001, all χ^2^_p_(1) = 16.69, p_corr_ < 0.001; χ^2^_n_(1) > 9.23, p_corr_ < 0.01. For both impulsivity and hyperactivity the group effect was significant for the first three school years (impulsivity: all χ^2^(1) > 8.36, p_corr_ <0.05) but not year 4 (χ^2^_year4_(1) = 2.52, *p* = 0.112; hyperactivity: all χ^2^(1) > 5.370, p_corr_ <0.05; χ^2^_year4_(1) = 0.170, *p* = 0.680). Furthermore, a significant difference was only evident when comparing first and 4th year (impulsivity: χ^2^_(year1> year4)_(1) = 6.694, *p* < 0.05; hyperactivity: χ^2^_year4_(1) = 10.602, p_corr.<_0.01) but not for the comparison of 1st to 2nd and 3rd year (impulsivity: χ^2^(1) < 0.9049, *p* > 0.330; hyperactivity: all χ^2^(1) > 3.019, *p* > 0.066).

### Subcriteria of inattention

**Fig. 3:**
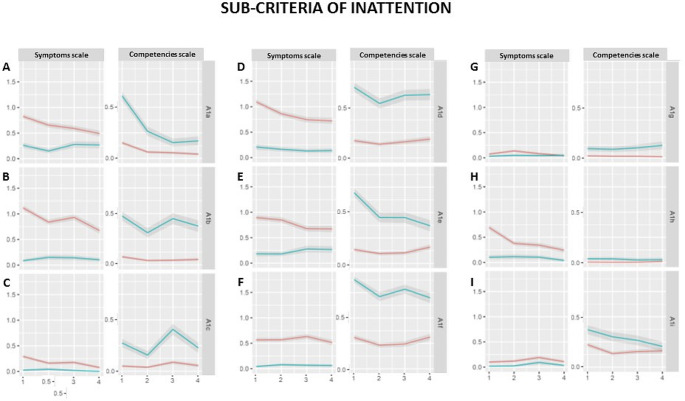
Subcriteria of attentiveness in primary school reports. The mean values of all ICD-10/DSM-5 subcriteria of inattention are shown on the symptoms scale and of the complementary competencies on the competencies scale. **(A)** A1a = “often fails to give close attention to details or makes careless mistakes in schoolwork, at work, or with other activities”/ “often pays attention to detail and rarely makes careless mistakes in schoolwork, work, or other activities”, **(B)** A1b = “often has trouble holding attention on tasks or play activities”/“is alert for extended periods of time when performing tasks or playing games”, **(C)** A1c = “often does not seem to listen when spoken to directly”/ “is an attentive listener and notices when others speak to him or her”, **(D)** A1d = “often does not follow throught on instructions and fails to finish schoolwork, chores, or duties in the workplace”/”completely carries out instructions and finishes schoolwork, other work, or duties in the workplace”, **(E)** A1e = “often has trouble organizing tasks and activities”/„organizes his tasks and activities effortlessly”, **(F)** A1f = “often avoids, dislikes, or is reluctant to do tasks that require mental effort over a long period of time”/ ” prefers, likes or tolerates to work on tasks that require sustained mental effort and is ambitious”, **(G)** A1g = “often loses things necessary for tasks and activities”/ ”has items needed for specific tasks and activities”, **(H)** A1h = “is often easily distracted”/ “is not distracted by external stimuli”and **(I)** A1i = “is often forgetful in daily activities”/ ”Is reliable in everyday activities”. Abscissa: school year (n). Ordinate: mean of operationalization of ICD-10/DSM-5 sub-criteria of inattention and of complementary competencies (n). For the symptoms scale, descriptions of 0 = no symptom in a school report according to semantic analysis, 1 = sub-clinical expression, 2 = clinical expression. For the competencies scale, descriptions of 1 = description of expressions of typically developed behavior complementary to the ADHD symptom, 0 = feature not mentioned. Red line: children with ADHD, blue line: typically developing children. Colored lines indicate group means and shaded ribbons indicate the standard error of means

As descriptions of inattention were much more frequent in school reports than those of the other main symptoms, ratings for all nine subcriteria of ICD-10/DSM-5 were performed and are visualized in Fig. 3. On a descriptive level, group differences can be observed for nearly all of the subcriteria, and appear to be most prominent for A1a (makes careless mistakes), A1b (has trouble in holding attention), A1d (fails to finish work), A1e (has trouble in organizing tasks) and A1f (avoids long-lasting tasks).

### Diagnostic performance and preliminary thresholds

**Fig. 4:**
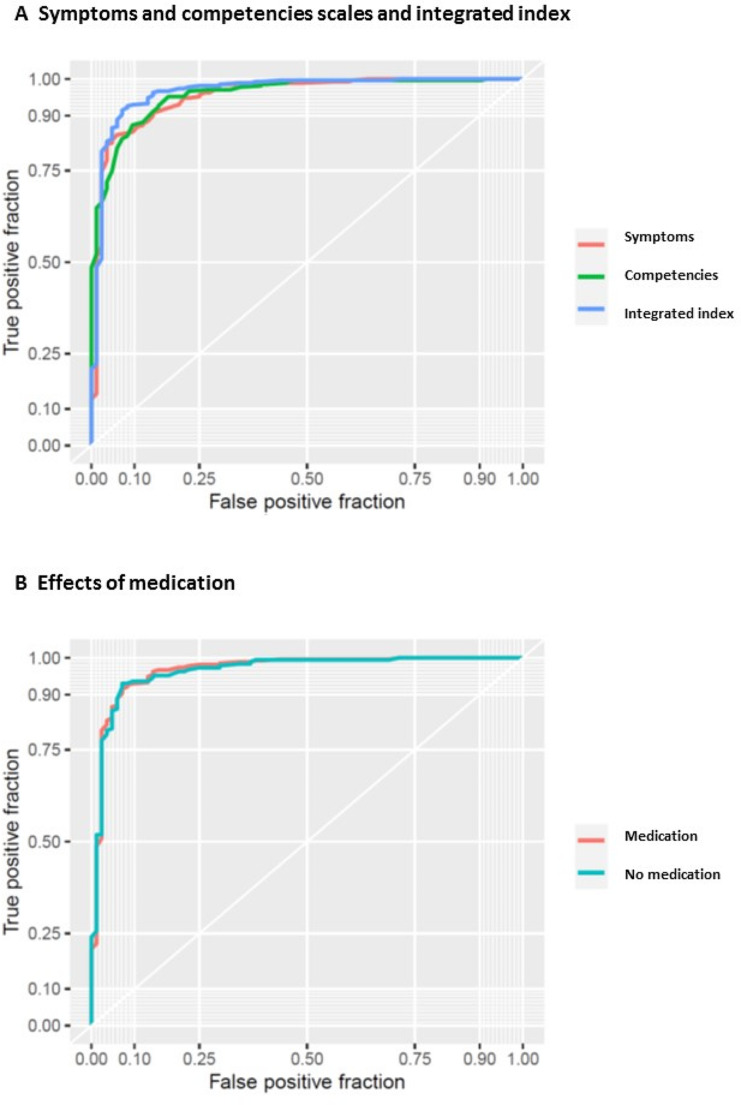
Sensitivity and specificity. Data are expressed as receiver operating characteristic (ROC) curves to visualize diagnostic accuracy and discrimination thresholds. **(A)** ROCs of symptoms and competencies scales and of integrated index. Area under the curve (AUC): AUC(symptoms): 0.96, AUC(competencies): 0.96, AUC(integrated index): 0.97. **(B)** ROCs of ADHD including patients with medication (mi) and ADHD without medication (nm) data. AUC(nm): 0.97 AUC(mi): 0.97

To further explore the discriminative value of symptoms and competencies scales and the integrated index and their potential as a diagnostic marker for retrospective diagnosis, we analyzed ROC curves and determined the area under the curve along with sensitivity and specificity as an index of diagnostic performance. Figure 4A depicts ROCs of the summary symptoms and competencies scales and the integrated index. This analysis revealed high values for the area under the curve (AUC): AUC(symptoms): 0.95, AUC(competencies): 0.96, AUC(integrated index): 0.97. Next, we determined preliminary diagnostic thresholds for (1) maximized balanced sensitivity and specificity, and (2) different levels of fixed specificity. Integrated index performed exceptionally in this regard, providing an estimated sensitivity of ∼ 0.91 and specificity of ∼ 0.93 with an optimized balanced threshold of 0.59 (see also Fig. 1B).

Further increasing the desired specificity to ∼ 0.95 (threshold 1.8) resulted in a decrease of estimated sensitivity to ∼ 0.83, while an increase of specificity to ∼ 0.99 (threshold 5.33) further decreased sensitivity to ∼ 0.33. The ROC curve and the area under the curve was hardly affected when school reports were included/excluded for patients that were medicated at this specific time point (Fig. 4B). For more details and performance of symptoms and competencies scales, respectively, see Table 2.

**Table 2:** Measures for diagnostic performance. Data are expressed with several optimized thresholds for balanced sensitivity (optimized for maximum youden index (i.e. specificity + sensitivity-1), or optimized for maximum sensitivity for a given fixed specificity)

Valence	Threshold	optimized for	Sensitivity	Specificity	AUC	Youden index
Symptoms	2.94	youden index	0.86	0.90	0.95	0.76
Competencies	1.93	youden index	0.88	0.88	0.96	0.76
Integrated index	0.59	youden index	0.91	0.93	0.97	0.84
Symptoms	3.31	Sensitivity (specificity fixed at ∼ 0.95)	0.82	0.95	0.95	0.79
Competencies	1.46	Sensitivity (specificity fixed at ∼ 0.95)	0.75	0.95	0.96	0.70
Integrated index	1.80	Sensitivity (specificity fixed at ∼ 0.95)	0.83	0.95	0.97	0.79
Symptoms	6.05	Sensitivity (specificity fixed at ∼ 0.99)	0.33	0.99	0.95	0.32
Competencies	0.99	Sensitivity (specificity fixed at ∼ 0.99)	0.52	0.99	0.96	0.51
Integrated index	5.33	Sensitivity (specificity fixed at ∼ 0.99)	0.33	0.99	0.97	0.32

Additionally, we have performed a statistical test to compare the respective ROC curves for differences (DeLong test for correlated ROCs). The results indicate that the ROC curves are not statistically different when comparing symptoms vs. competencies (Z = 0.322, *p* = 0.747) and integrated index vs. competencies (Z = 1.019, *p* = 0.308), but a statistically significant difference could be observed for the comparison of integrated index vs. symptoms (Z = 2.711, *p* = 0.00671, pcorr. < 0.05), allowing for the conclusion of an added benefit for the integrated index measure as compared to just the symptoms scale measure.

To summarize, these preliminary cut-off scores suggest that a difference between summary symptoms and competencies scales above 0.59 (averaged across all school years) would indicate childhood ADHD retrospectively with high sensitivity and specificity (with respect to a TDC group). The symptoms and competencies cut-off scores alone would suggest ADHD above a mean summed symptoms scale of 2.94 or below a mean summed competencies scale of 1.93, respectively.

## Discussion

The current study presents a new systematic approach to use descriptions of academic and social behaviors in German school reports to characterize ADHD criteria according to their definitions in ICD-10 and DSM-5, and explores its potential as a retrospective diagnostic marker for ADHD. All main criteria of ADHD were reflected in descriptions from school report data, with particular emphasis on inattention and its subcriteria. All analyzed ICD-10/DSM-5 main criteria of ADHD showed highly significant differences between children with and without ADHD across school years, with higher discrimination for earlier school years, and particular strong differences during the first school year. The clinical symptomatology of ADHD is defined in current nosologies by inattention, hyperactivity and impulsivity, i.e. descriptions of impairments of the three behavioral dimensions attentiveness, activity and impulsivity/self-control. Analyzing primary school certificates, we were able to assess also competencies in these dimensions systematically for the first time and show their additional potential for differentiating typical and atypical behavior related to a diagnosis of ADHD. This is a remarkable and clinically relevant finding, suggesting that clinical evaluation of school reports in current clinical practice should not only focus on descriptions reminiscent of symptoms, but also on the *lack of described competencies* for adaptive behavior in the domains of attention, calmness, and self-control (Figs. 1, 2 and 3).

There was a higher discrimination for ADHD in the descriptions of academic performance and social behaviors in earlier school reports. This could result from the more extensive and detailed respective state requirements for first and second grade of primary school in Saxony. Another explanation could be changes in ADHD symptoms across age [7]. Nevertheless, the finding emphasizes the value of qualified descriptions of patients’ behaviors in school reports for the diagnostic process in general, potentially not restricted to ADHD.

With regard to the existing literature, this is to our knowledge the first study to quantitatively examine ADHD symptoms in primary school reports. This is surprising as the assessment of school reports is explicitly recommended in clinical guidelines, including the assessment of childhood ADHD [12–14, 38]. In line with a general approach to study quantitative empirical evidence of retrospective clinical information, our group has recently published quantitative analyses of family and developmental history in patients with ADHD and with autism spectrum disorder [29, 42]. However, the retrospective quantitative analysis of ADHD symptoms in school reports appears to address a particularly relevant and currently unsolved clinical problem, which is central to the diagnostic challenge in adolescents and adults.

Although there is a large body of evidence about ADHD in the general context of school, the medical literature on ADHD is limited with respect to school reports. There are the established textbook knowledge and ICD-10/DSM-5 descriptions about the behavior of children with ADHD in school [2, 3, 16]. Recent papers reporting on the behavior of children with ADHD in school neither added new descriptions to the established ADHD syndrome, nor investigated school reports in any systematic manner [42–44]. Similarly, the literature from pedagogical and educational sciences does not provide additional scholarly evidence here [44].

Importantly, our study demonstrates the feasibility for constructing a novel quantitative diagnostic instrument to solve the problem of retrospective diagnosis of childhood ADHD in adolescence and adulthood. Currently, two main alternatives are used for retrospective analysis of childhood ADHD. First, full clinical and psychosocial assessment is the primary method according to the clinical guidelines [12–14, 38] but does not include any specific strategies for the systematic assessment of retrospective information, thus, it is up to the individual experience and expertise of the clinician to decide what types of data are considered. Consequently, it is hard to evaluate the accuracy of this strategy empirically, because the retrospective assessment is a qualitative aspect of the clinical evaluation itself. The second alternative, self-rating instruments, have been evaluated for diagnostic accuracy, and several studies have called into question their validity [24–27]. Importantly, both strategies suffer from potential bias, an inherent confound for each retrospective assessment. Quantitative analysis of school reports may solve this problem in an elegant way and appears to be a unique and promising strategy: The assessment of the patient was performed by a professional (i.e. a teacher) in the past, but can be “re-evaluated” today for its content with respect to indications of ADHD.

Assessing potential diagnostic accuracy of our approach using ROC curves (Fig. 4), we found strong sensitivity and specificity for symptoms and competencies scales and integrated index, and no strong effect of medication. These findings underline the potential of this approach to design a novel diagnostic tool. In a real-world clinical scenario, the clinician would strongly benefit from an algorithm how to assess school reports for ADHD symptoms and how to use cut-off values in order make a diagnostic decision. In order to further assess the feasibility of this approach, we calculated potential cut-off values (Table 2). Translating these calculations into a clinically more sizeable unit, two strong descriptions of ADHD symptoms in a school report (corresponding to a summarized symptoms score of 4) would suggest childhood ADHD. In turn, two descriptions of explicitly adaptive behavior (corresponding to a summarized competencies score of 2) would suggest absence of childhood ADHD.

Together, we have shown here proof-of-principle that quantitative assessment of primary school reports is a valid approach to retrospectively assess childhood ADHD and has the potential to be used as a diagnostic marker. Our study indicates that an integrated index, integrating assessment of symptoms and competencies scales for all nine sub-criteria of inattention, and two overall criteria for hyperactivity and impulsivity, respectively, may provide an excellent distinction between children with ADHD and typically developing children. Importantly, assessing descriptions of competencies seems to add further discriminative power within our approach.

### Limitations and future directions

Albeit preliminary, this is the first study devising a quantitative diagnostic instrument (based on semantic information in school reports) for retrospective assessment of ADHD criteria minimizing potential memory biases, demonstrating very promising diagnostic accuracy. Some limitations need to be considered to evolve this approach further towards a general diagnostic instrument: The study was performed in the German federal state of Saxony and replications across Germany and internationally, exploring varied types of school reports, are necessary. In our sample 36% of the students received their diagnosis of ADHD in primary school age (until nine years). Potentially their parents could have informed the teachers about the diagnosis which could have influenced the teacher’s descriptions in some cases. School reports of children with other diagnoses than ADHD need to be included in future studies, to assess the specificity of our approach in the context of differential diagnoses. Lastly, analyzing academic performance and social behaviors in school reports with respect to a number of distinct rating scales takes time, needs clinical experience in the field of ADHD and may also require explicit training on the instrument itself. Thus, future studies should explore the potential of machine learning methods in the field of natural language processing for a direct translation of school reports into ADHD criteria profiles. Our findings, validating the feasibility of clinically valid symptom assessment are an important first step towards such an approach.

## Conclusion

This is the first study to quantitatively explore descriptions of ADHD in primary school reports. Strong differences between school reports from children with ADHD and with typically developing children suggest a novel and valid strategy to retrospectively assess childhood ADHD, which is currently a core challenge in the diagnostic process of ADHD in adolescents and adults.

## Electronic supplementary material

Below is the link to the electronic supplementary material.

Supplementary Material 1

Supplementary Material 2

Supplementary Material 3
