# Supplementary material for: Retrospective assessment of ICD-10/DSM-5 criteria of childhood ADHD from descriptions of academic and social behaviors in German primary school reports
Source: Eur Child Adolesc Psychiatry. 2024 Jul 24;34(2):659–73. doi: 10.1007/s00787-024-02509-4 (PMC11868344; doi:10.1007/s00787-024-02509-4)
Supplement: Supplementary file 1 — Supplementary Material 1 [file 787_2024_2509_MOESM1_ESM.pdf]

Supplementary Table 2: Examples of behavioral descriptions in primary school reports

Symptoms					Competencies		
**ICD-10/DSM-V**		**Clinical Criteria**<br>**2**	**Subclinical Criteria**<br>**1**	**Not Mentioned**<br>**0**	**Typical Development**<br>**1**		
**1. Description of Inattention**							**1. Description of Attention**
1. A) Fails to give close attention to details or makes **careless mistakes** in schoolwork, work or other activities.	e. g. overlooks or omits details, works inaccurately.	*"He has trouble writing correctly."*	*"If he tries, he doesn't make as many careless mistakes and he improved his writing performance."*		*"He writes according to standards and pays attention to details."*	e. g. pays attention to details and works precisely and according to standards.	1. A) Often pays attention to detail and **rarely makes careless mistakes** in schoolwork, work, or other activities.
1. B) Has trouble **sustaining attention** in taks or play activities.	e.g. has difficulty staying focused during classes, lectures, conversations, prolonged reading.	*"He is not able to concentrate properly in class"*	*"At times he finds it difficult to concentrate."*		*"He works concentrated and focused in class."*	e.g. stays focused during class, lectures, conversations, or prolonged reading.	1. B) Is **alert for extended periods of time** when performing tasks or playing games.
1. C) Often **does not seem to listen** when spoken to directly.	e.g. seems to be elsewhere with his mind, even with no obvious distractions.	*"He can rarely answer questions because he dreams a lot in class"*	*"Sometimes he seems absent in class, but he is improving his behavior."*		*"He participates actively in discussions in class and has good answers to questions."*	e.g. follows the lesson actively and listens carefully.	1. C) Is an **attentive listener** and notices when others speak to him or her.
1. D) Does not follow through on instructions and **fails to finish** schoolwork, chores, or duties in the workplace (not due to oppositional behavior or failure to understand instructions.	e.g. starts tasks but quickly loses focus and is easily distracted.	*He rarely finishes his tasks, shows little perseverance and often needs help to continue working.*	*"In some subjects, he does his work incompletely and shows reduced persverance."*		*"He completes his tasks persistently and carefully."*	e.g. starts with tasks, keeps focus and completes them.	1. D) Completely carries out instructions and **finishes** schoolwork, other work, or duties in the workplace.
1. E) Has difficulty **organizing** tasks and activities.	e. g. has trouble completing sequential tasks; difficulty keeping materials and belongings in order; untidy, haphazard, disorganized work; bad time management; fails to meet appointments and deadlines.	*"He has a chaotic working style and often makes subsequent mistakes, and he also submits tasks too late."*	*"He got along better with his learning and working materials, his working management was a little clearer and he only occasionally handed in tasks too late."*		*"He got along well with his learning and working materials, his bookkeeping is well structured and he submits his tasks on time."*	e.g. handles the following tasks sequentially; keeps materials and personal belongings in order, orderly and planned work, good time management, meets appointments and deadlines.	1. E) **Organizes** his tasks and activities effortlessly.
1. F) **Avoids, dislike or is reluctant** to engage in tasks that require **sustained mental effort** (such as schoolwork or homework).	e.g. avoiding participation in class or homework.	*"Too often he does something completely different in class than the class. It would be important for him to also work on unpleasant tasks."*	*"He already works a lot in class, sometimes he tries to avoid strenuous tasks."*		*"He works ambitious in class and does his homework conscientiously."*	z. B. macht begeistert im Unterricht mit und macht die Hausaufgaben mühelos und ehrgeizig	1. F) **Prefers, likes or tolerates to work** on tasks that require **sustained mental effort** and is ambitious.
1. G) **Loses things** necessary for tasks or activities.	e.g. lack of school supplies, pens, books, tools, purses, keys, work papers, glasses, mobile phones.	*"He often cannot start work because his work materials are missing. Even in sports he has to bring his sports bag."*	*"Occasionally his working materials are incomplete."*		*"His working materials are complete and always ready for use."*	e.g. having school supplies, sports bags, pens, books, tools, purses, keys, work papers, glasses, mobile phones.	1. G) **Has items** needed for specific tasks and activities.
1. H) Is **easily distracted** by extraneous activities.	e.g. distractability, including thoughts unrelated to the current situation.	*"He is easily distracted and is easily disturbed by his classmates."*	*"Sometimes his is distracted by his classmate next to him."*		*"He works in a concentrated and goal-oriented manner, even under disruptive factors."*	e.g. works in a focused manner and is not distracted by external stimuli or distracting thoughts.	1. H) Is **not distracted** by external stimuli.
1. I) Is often **forgetful** in daily activities.	e.g. forgetting to do errands or keep to appointments or class duties.	*"He regularly forgets his class duties and unreliably completes assigned tasks."*	*"Sometimes he forgets his classwork or is late from lunch."*		*"He carries out the tasks assigned to him reliably and conscientiously."*	e.g. reliable in completing household chores and errands or keeping appointments or class duties.	1. I) Is **reliable** in everyday activities .

Supplementary Table 2: Examples of behavioral descriptions in primary school reports

2. Description of Hyperactivity							2. Description of age-appropriate Activity
**Excessive** and **age-inappropriate motor activity.**	e.g. difficulty sitting still, inappropriate to the situation, tipping, wriggling with arms and legs, excessive walking or climbing, constant movement.	*"He is so restless and fidgety in class that his classmates can hardly work next to him."*	*"Sometimes he still wobbles in his chair, but he refrains from doing after request."*		*"He works calmly."*	e.g. sits calmly in his place, has a calm working attitude.	**Age-appropriate motor activity.**
**3. Description of Impulsivity**							**3. Description of Age-appropriate interaction behavior**
**Excessive** and **age-inappropriate impulsive interaction.**	e.g. B. has difficulty playing or working quietly, talks excessively, often blurts out answers, has difficulty waiting for turn, often interrupts and disrupts others, fails to follow discussion rules in class.	*"He is very loud and restless in class. He needs to learn to speak up and abide by our rules of conversation."*	*"He has already calmed down in class and only sometimes calls in the answer without answering."*		*"He has internalized our discussion rules and waits his turn."*	e.g. plays quietly, does not speak excessively, can wait for turn, listens and does not interrupt, can follow rules of discussion in class.	**Age-appropriate** calm interaction behavior.

Supplementary Table 2: Examples of behavioral descriptions in primary school reports

The table includes exemplary descriptions from primary school reports related to student behavior. On the left is the clinical symptom description according to ICD-10/DSM-V for inattentiveness, hyperactivity and impulsivity. If the criterion is fully met, it is rated as a clinical marker (- 2). If the criterion is only partially met or is in remission, it is rated as a subclinical marker (- 1). On the right side there is the competency description as a counterpart to the ICD-10/DSM-V criteria for attention, age-appropriate motor behavior and age-appropriate interaction behavior (1). If the criterion is not mentioned in the school report, it is rated as (0). If the school report is missing, it is marked as missing data (/). Only one category (clinical, subclinical or typical development or not mentioned) can be assigned per half-year report and criterion.
