# Supplementary material for: Retrospective assessment of ICD-10/DSM-5 criteria of childhood ADHD from descriptions of academic and social behaviors in German primary school reports
Source: Eur Child Adolesc Psychiatry. 2024 Jul 24;34(2):659–73. doi: 10.1007/s00787-024-02509-4 (PMC11868344; doi:10.1007/s00787-024-02509-4)
Supplement: Supplementary file 3 — Supplementary Material 3 [file 787_2024_2509_MOESM3_ESM.pdf]

# Supplementary Figure 1: Schools, teachers and school certificates in Dresden, Saxony

## 1. Map symbols

| Number of school certificates | Triangle colour |
|---|---|
| 1-10 | |
| 11-20 | |
| 21-30 | |
| 31-40 | |
| 41-50 | |
| 51-60 | |
| 61-70 | |
| 71-80 | |
| 81-90 | |
| 91-100 | |

*Triangle:*
Control students

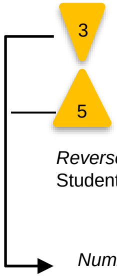


*Reverse triangle:*
Students with ADHD

*Number of teachers*

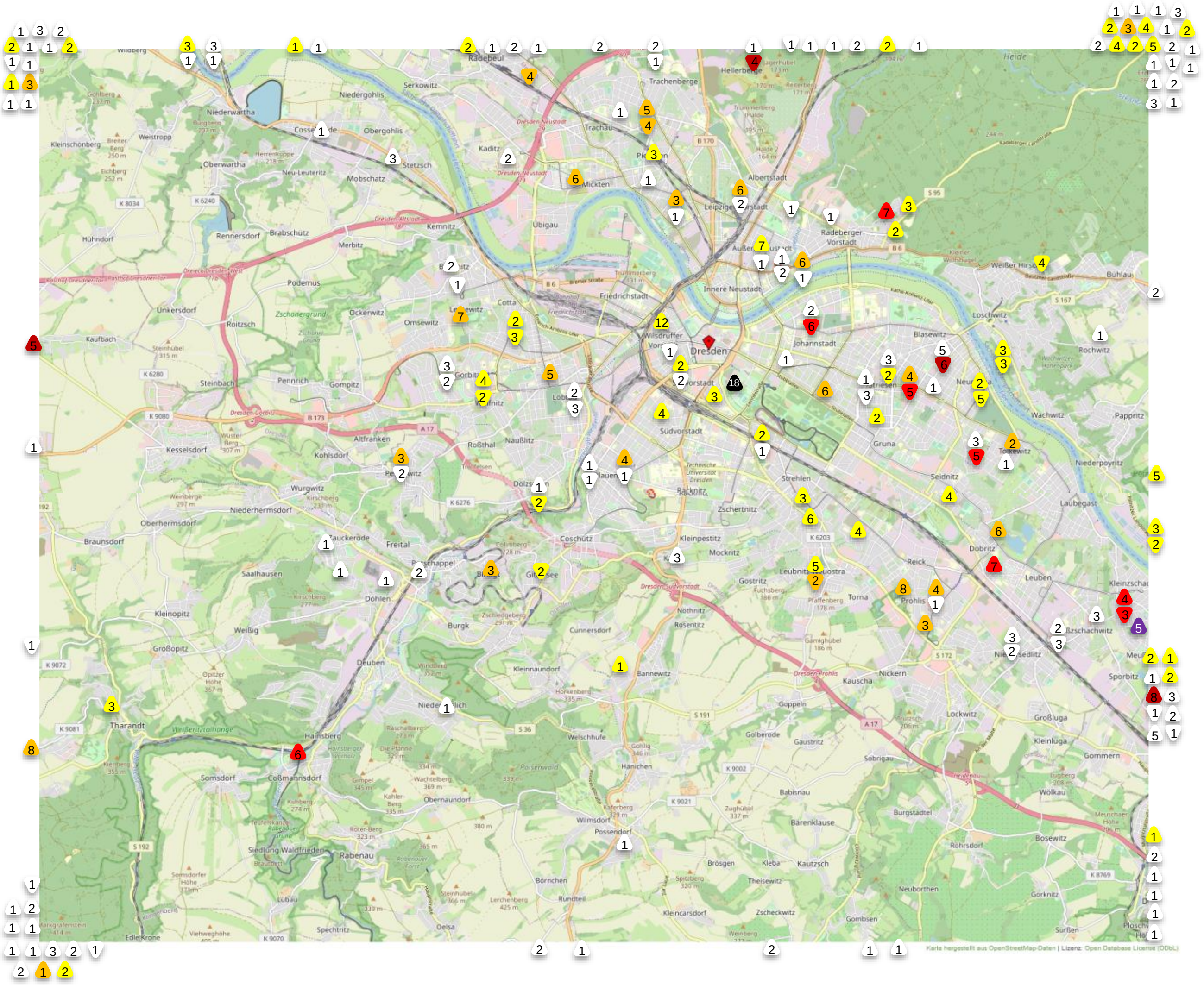

| 2. Numbers of… | …schools | …teachers | …school certificates |
|---|---|---|---|
| ADHD | 173 | 433 | 1197 |
| control | 53 | 118 | 656 |
| total | 191 | 545 | 1853 |

| 3. Year of enrollment | | 2001/ 2002 | 2002/ 2003 | 2003/ 2004 | 2004/ 2005 | 2005/ 2006 | 2006/ 2007 | 2007/ 2008 | 2008/ 2009 | 2009/ 2010 | 2010/ 2011 | 2011/ 2012 | 2012/ 2013 | 2013/ 2014 | 2014/ 2015 |
|---|---|---|---|---|---|---|---|---|---|---|---|---|---|---|---|
| Students | ADHS | 1 | 2 | 6 | 8 | 18 | 18 | 20 | 22 | 28 | 30 | 31 | 35 | 24 | 19 |
| | control | 2 | 1 | 4 | 4 | 4 | 10 | 10 | 16 | 15 | 13 | 9 | 6 | 1 | 0 |
| | total | 3 | 3 | 10 | 12 | 22 | 28 | 30 | 38 | 43 | 43 | 40 | 41 | 25 | 19 |

**Supplementary Figure 1: Schools, teachers and school certificates in Dresden**

Subtable 1 shows the map symbols for Dresden city.
Subtable 2 shows the numbers of schools, teachers and school certificates.
Subtable 3 shows the year of student enrollment.
